# Energy and Charge Transport in 2D Atomic Layer Materials: Raman-Based Characterization

**DOI:** 10.3390/nano10091807

**Published:** 2020-09-10

**Authors:** Ridong Wang, Tianyu Wang, Hamidreza Zobeiri, Dachao Li, Xinwei Wang

**Affiliations:** 1 State Key Laboratory of Precision Measuring Technology and Instruments, Tianjin University, Tianjin 300072, China; rdwang@tju.edu.cn; 2 Institute of Chemistry, Chinese Academy of Sciences, Beijing 100190, China; tywang@iccas.ac.cn; 3 Department of Mechanical Engineering, Iowa State University, Ames, IA 50011, USA; hzobeiri@iastate.edu

**Keywords:** 2D materials, energy transport, charge transport, Raman spectroscopy

## Abstract

As they hold extraordinary mechanical and physical properties, two-dimensional (2D) atomic layer materials, including graphene, transition metal dichalcogenides, and MXenes, have attracted a great deal of attention. The characterization of energy and charge transport in these materials is particularly crucial for their applications. As noncontact methods, Raman-based techniques are widely used in exploring the energy and charge transport in 2D materials. In this review, we explain the principle of Raman-based thermometry in detail. We critically review different Raman-based techniques, which include steady state Raman, time-domain differential Raman, frequency-resolved Raman, and energy transport state-resolved Raman techniques constructed in the frequency domain, space domain, and time domain. Detailed outlooks are provided about Raman-based energy and charge transport in 2D materials and issues that need special attention.

## 1. Introduction

Owing to their extraordinary electrical, optical, and mechanical properties, two-dimensional (2D) materials, for instance, graphene, black phosphorus, hexagonal boron nitride, transition metal dichalcogenides (TMDs), and MXenes, can be used in many different areas, for instance, optoelectronic devices, biosensing, energy storage, etc. [1,2,3,4,5,6,7]. Furthermore, the thermal transport, interface thermal transport, electronic structures, physical structures, and hot carrier transport properties of these 2D materials are of great importance in fundamental research, as well as in engineering applications [8]. From the perspective of fundamental research, it is of great importance to explore the physics behind thermal dissipation and thermal management at the micro/nanoscale. For application, with the rapid increase in power density in modern electronics, the heat accumulation becomes a bottleneck for further miniaturization. As the heat accumulation in electronic and optoelectronic devices raises the operating temperature, the device performance and lifetime can be influenced. Thus, it is in high demand to improve the heat conductance and reduce the interface thermal resistance [8,9].

Over the last few years, many simulation-based methods have been reported to characterize the thermal transport in 2D materials, such as molecular dynamics simulation, non-equilibrium Green function method, the Boltzmann transport equation, and the first-principles-based multi-temperature model [10,11,12,13]. Many experimental methods, such as time-domain thermoreflectance (TDTR), the microbridge method, the 3ω method, the laser flash technique, and Raman spectroscopy, are employed to explore the thermal properties of 2D materials [14,15,16,17]. For TDTR, it requires complicated setups and careful operation. The accuracy of the microbridge method is affected by the thermal contact resistance between the sample and contact, and by the difficulty in evaluating the tiny heat flow sustained by very thin samples. The 3ω method is vulnerable to the harmonic noises in the current source. The laser flash technique will become extremely difficult to use for measuring very thin samples (a few µm) and for measurement at cryogenic temperatures. Raman spectroscopy, which carries signature information about materials regardless of their distance and size, provides a unique way of looking into the energy transport, hot carrier diffusion, and physical structure of 2D materials. As a noncontact optical method, Raman-based thermometry is able to realize precise (material specific) and specific thermal properties characterization of 2D materials with sub-micron size by focusing the excitation laser to a very small spot.

Currently, many novel and new Raman-based techniques have been developed to meet different requirements of 2D materials measurement. For instance, different energy transport states are constructed to study the thermal conductivity, hot carrier diffusion, and interface thermal resistance of suspended or supported 2D materials. In the following sections, a comprehensive critical review of various Raman-based techniques developed for energy and charge transport in 2D materials is presented to give a clear picture of the progress in this field. In addition, potential research perspectives in the field of 2D materials using these Raman-based techniques are also discussed.

## 2. Steady State Raman

For steady state Raman, an electrical current or a continuous-wave laser is applied to the 2D materials to realize steady state heating. Meanwhile, the sample is irradiated by an excitation laser and the corresponding Raman signal is collected. Based on the temperature-dependence characteristic Raman signal, the interface resistance between the sample and the substrate or the thermal conductivity of the sample can be determined.

**Figure 1:**
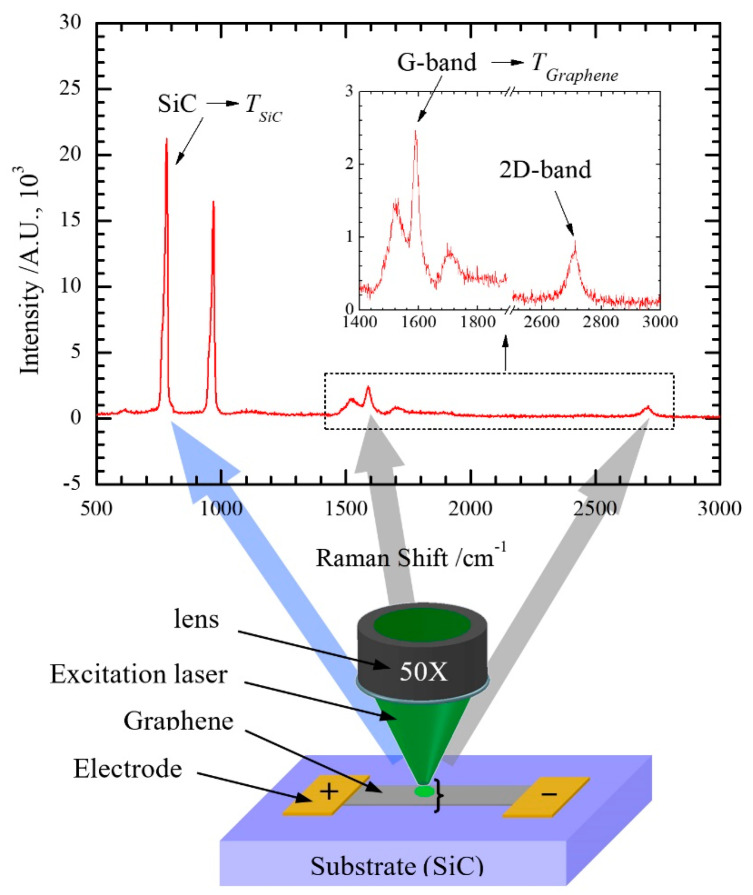
Illustration of the sample under electrical heating for measuring interface thermal resistance. The top figure depicts the Raman spectrum of epitaxial graphene on 4H-SiC. Reproduced from [18], with permission from John Wiley and Sons.

**Figure 2:**
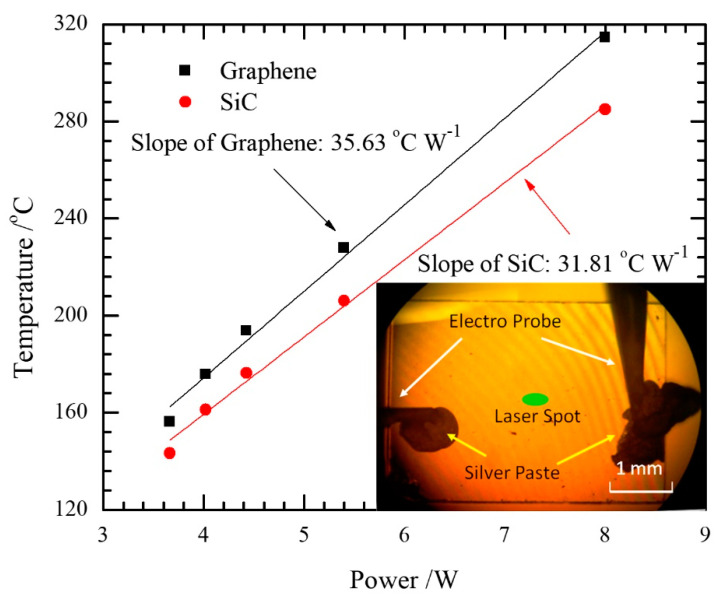
The relationship between temperature and heating power. Reproduced from [18], with permission from John Wiley and Sons.

Yue et al. developed an electrical heating method for the interfacial thermal resistance measurement of epitaxial graphene on 4H-SiC [18]. As shown in Figure 1, the steady state heating of the sample is achieved with an electrical current passing through. Furthermore, a confocal Raman system is used to obtain the Raman signal with an excitation laser irradiating the graphene. Then, the temperature of graphene and SiC can be differentiated based on the corresponding Raman signals. Finally, the interfacial thermal resistance between these two materials can be derived based on $R_{\mathrm{tc}}=(T_{\mathrm{graphene}}-T_{\mathrm{SiC}})\cdot A/(I^{2}\cdot R)$, where *A* is the graphene area, *R* is the electrical resistance of graphene, and *I* is the applied current. Due to the large uncertainty originated from a single temperature point, a linear fitting of the relation between the temperature and input power of Joule heating is conducted. The equation for determining thermal resistance can be rewritten as $R_{\mathrm{tc}}=(T_{\mathrm{graphene}}^{\prime }-T_{\mathrm{SiC}}^{\prime })\cdot A$, where $T_{\mathrm{graphene}}^{\prime }$ and $T_{\mathrm{SiC}}^{\prime }$ are the temperature against input power slopes shown in Figure 2.

**Figure 3:**
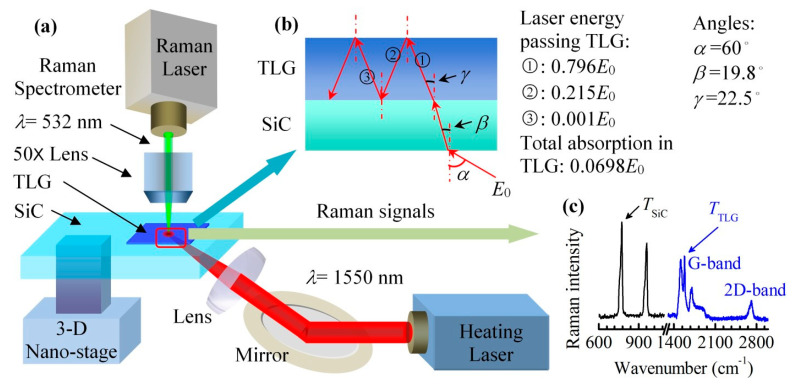
(**a**) Experimental system used for characterizing the tri-layered graphene/SiC interface. An infrared laser is used to heat the sample and a 532 nm laser is used to excite Raman signals. (**b**) Laser propagation and accumulated energy transmission. (**c**) Raman spectra of graphene and SiC. Reproduced from [20] with permission from The Royal Society of Chemistry.

Though the heating level can be controlled accurately by adjusting the electrical current, the results can be affected by the contact resistance between the electrode and the sample. Additionally, precise positioning of the laser is also important for Raman-based temperature measurement. To overcome these drawbacks, Tang et al. developed a dual laser Raman-based thermal probing method with a superior spatial resolution [19,20]. In this method, two lasers are used: one is for thermal probing, and the other one is for heating. In this work, the interfacial energy coupling across graphene/substrate interfaces is characterized, and the experimental setup is shown in Figure 3. The sample is placed on a nanostage, which is controlled by a piezoelectric actuator. As the stability is improved dramatically and the positioning resolution could be down to as small as 5 nm, the noise level in Raman spectra is greatly reduced.

During calibration and real interface measurement, 2D materials experience different stress effect. In calibration, the temperatures of the 2D material and the substrate are the same. However, the temperature of the 2D material is higher than the substrate in actual experiment. Additionally, the 2D material has different thermal expansion coefficient from the substrate. Therefore, it does not mean the temperature is determined precisely in experiment, even with high quality calibration. Furthermore, during experiment, the local spacing at the interface will significantly affect the laser absorption, which could probably lead to very high error in laser absorption calculation. All these issues need to be resolved in order to obtain high-level understanding of the energy transport in 2D materials interface. Raman shift, which is related to temperature and stress, has a higher sensitivity to temperature than Raman linewidth. Based on this, for the first time, Tang et al. decoupled the thermal and mechanical behavior by looking into the difference in temperature determined by using Raman shift and linewidth [20], that is, the local stress effect is extracted, and the interface spacing effect is also evaluated based on the corrugation-induced Raman enhancement.

Furthermore, Yuan et al. also studied the interface thermal conductance between few to tens-layered molybdenum disulfide (MoS_2_) and crystalline silicon (c-Si) [21]. In this work, only one laser, which is for both thermal probing and heating, is used. Furthermore, it is proved that there is a spacing between MoS_2_ and c-Si, which can lead to a much lower interfacial thermal conductance. As the thermal expansion coefficients of MoS_2_ and c-Si are different, the imperfect contact between MoS_2_ and c-Si could become much smoother after laser heating. In addition, with the increased sample thickness, the mechanical stiffness is improved and a better interface contact between MoS_2_ and c-Si is obtained. Thus, the interface thermal conductance increases. In short, the interface spacing effect is a very crucial factor in studying the interfacial thermal conductance, and it is necessary to further investigate thermal expansion coefficients of the sample and substrate. Zobeiri et al. studied the thermal expansion coefficient of WS_2_ in detail [22]. In this work, the in-plane and cross-plane linear thermal expansion coefficients of WS_2_ were considered separately. Furthermore, the in-plane linear thermal expansion coefficient, which plays a very important role in calculating the theoretical air gap thickness between WS_2_ and Si substrate, was determined. The range is about 5 × 10^−6^ to 8 × 10^−6^ K^−1^ with temperatures varying from 300 to 700 K, and agrees well with reference values.

## 3. Time-Domain Differential and Frequency-Resolved Raman

For steady-state Raman spectroscopy, a relationship between temperature and Raman signal is needed. Both temperature calibration and laser absorption measurement are required to ensure the accuracy of the measurement. However, the temperature calibration is very time-consuming and could bring about large errors. Due to unknown optical property variation of different samples, the laser absorption measurement can also introduce very large errors. To overcome these critical physics problems faced in steady-state Raman spectroscopy, techniques involving time resolving will be more appreciated.

### 3.1. Time-Domain Differential Raman (TD-Raman)

**Figure 4:**
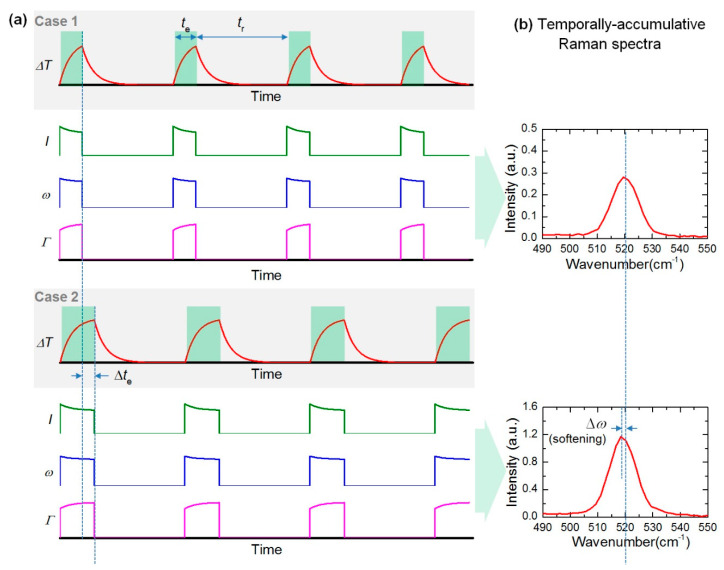
(**a**) The temperature evolution of the sample, and corresponding variations of Raman peak intensity (*I*), peak shift (*ω*) and linewidth (*Γ*). (**b**) Temporally accumulative Raman spectra of one laser pulse cycle in Case 1 and Case 2. Reprinted from [23], with permission from © The Optical Society.

The TD-Raman technique is inspired by the transient electro-thermal (TET) technique, which is developed for effective thermal characterization of one-dimensional (1D) materials [23,24]. In this technique, a single modulated laser beam is used for both sample heating and thermal probing. The concept of this technique is shown in Figure 4a. Both transient and steady-state measurements are conducted for this technique. The transient measurement consisted of an excitation period (*t*_e_) and a thermal relaxation period (*t*_r_). Furthermore, *t*_r_ is required to be long enough for the sample to completely cool down upon coming of next laser cycle. Multiple cycles are used to collect sufficient Raman signal. 

As shown in Figure 4a, during the excitation period, there is a temperature rise and Raman signal is also collected. With the increase of sample temperature, the Raman intensity decreases, the peak is redshifted and the linewidth broadens. During *t*_r_, as the laser is turned off, no Raman signal is collected in this period. Comparing the two cases, the excitation period in Case 2 is a bit longer than Case 1, while the thermal relaxation periods are the same. Due to the longer excitation time, the temperature increases further, and the corresponding Raman spectrum also varies. Figure 4b shows the temporally accumulative Raman spectra in the two cases. It can be seen that the Raman intensity in Case 2 is higher than that in Case 1, and a Raman peak position softening is observed. By combining the above Raman signal variation and further physical data analysis, the temperature evolution of the sample can be obtained to determine the thermal diffusivity of the sample [23].

In the work of TD-Raman development, the thermal diffusivity of a Si cantilever is measured. In the experimental setup, one end is connected to bulk base and the other end is heated by a modulated laser. As the length of the sample is much larger than the diameter or thickness of the sample, a one-dimensional (1D) model is used for the heat conduction with the governing equation as:

(1) $$\rho c_{p}\frac{\partial \Delta T}{\partial t}=\kappa \frac{\partial^{2}\Delta T}{\partial x^{2}}+q_{0},$$

where *ρ* is the density, *c_p_* is the specific heat of the sample, ΔT is the temperature rise, *κ* is the thermal conductivity, *x* is the distance away from the heat sink, and *q*_0_ is the heat generation per unit volume induced by the laser heating. The spatially averaged temperature rise can be obtained based on the following equation:

(2) $$\bar{\Delta T}(t)=\frac{2q_{0}L^{3}}{\kappa (x_{2}-x_{1})}\sum_{m=1}^{\infty }\frac{1}{m^{4}\pi^{4}}\left[1-\exp(-\frac{m^{2}\pi^{2}\alpha t}{L^{2}})\right]{\left(\cos\frac{m\pi }{L}x_{1}-\cos\frac{m\pi }{L}x_{2}\right)}^{2},$$

where *α* is the thermal diffusivity, and *L* is the sample length. The normalized temperature rise is then obtained as:

(3) $${\bar{\Delta T}}^{*}=\frac{\bar{\Delta T}(t)}{\bar{\Delta T}(t\to \infty )}=\frac{\sum_{m=1}^{\infty }\frac{1}{m^{4}\pi^{4}}\left[1-\exp(-\frac{m^{2}\pi^{2}\alpha t}{L^{2}})\right]{\left(\cos\frac{m\pi }{L}x_{1}-\cos\frac{m\pi }{L}x_{2}\right)}^{2}}{\sum_{m=1}^{\infty }\frac{1}{m^{4}\pi^{4}}{\left(\cos\frac{m\pi }{L}x_{1}-\cos\frac{m\pi }{L}x_{2}\right)}^{2}}.$$

As the variations of Raman signal are linearly related to temperature rise, the Raman spectra obtained in experiment are employed to determine the average temperature rise of the sample in the heated area. Furthermore, the accumulative Raman emission for one excitation cycle (from 0 to *t*_e_) is calculated based on the equation below:

(4) $$E_{\omega }(\omega ,t_{e})=I_{0}\int_{0}^{t_{e}}(1-A{\bar{\Delta T}}^{*})\exp\left[-\frac{4\ln2\cdot {\left(\omega -\omega_{0}+B{\bar{\Delta T}}^{*}\right)}^{2}}{{\left(\Gamma_{0}+C{\bar{\Delta T}}^{*}\right)}^{2}}\right]dt,$$

where *I*_0_, *ω*_0_, *Γ*_0_ are the corresponding Raman properties at the beginning of laser heating, *A*, *B*, *C* are the changing rate of Raman intensity, Raman shift, and linewidth against the normalized temperature. Then, a parameter named Fourier number *Fo* (*Fo* = *α*t/*L*^2^) is substituted into Equation (4) to get the following equation:

(5) $$E_{\omega }(\omega ,Fo_{e})=I_{0}\int_{0}^{Fo_{e}}(1-A{\bar{\Delta T}}^{*})\exp\left[-\frac{4\ln2\cdot {\left(\omega -\omega_{0}+B{\bar{\Delta T}}^{*}\right)}^{2}}{{\left(\Gamma_{0}+C{\bar{\Delta T}}^{*}\right)}^{2}}\right]dFo,$$

where *Fo*_e_ = *αt*_e_/*L*^2^. In order to eliminate the integration time’s effect, a normalized intensity is used and the expression is:

(6) $$E_{\omega }^{*}(\omega ,Fo_{e})=\frac{I_{0}}{Fo_{e}}\int_{0}^{Fo_{e}}(1-A{\bar{\Delta T}}^{*})\exp\left[-\frac{4\ln2\cdot {\left(\omega -\omega_{0}+B{\bar{\Delta T}}^{*}\right)}^{2}}{{\left(\Gamma_{0}+C{\bar{\Delta T}}^{*}\right)}^{2}}\right]dFo.$$

**Figure 5:**
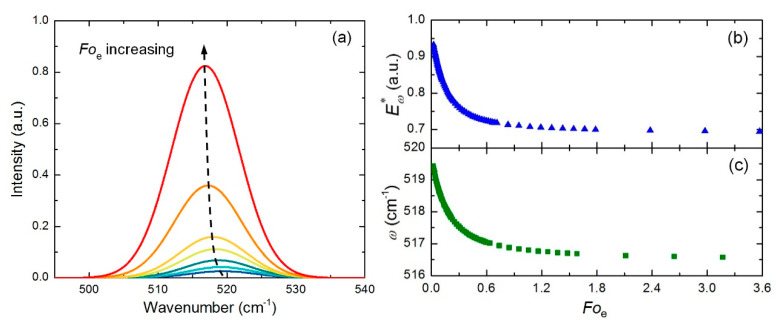
(**a**) The relationship between the reconstructed silicon Raman spectrum per cycle and the Fourier number. (**b**) The decreasing trend of the normalized Raman intensity against the Fourier number. (**c**) The decreasing trend of the Raman shift against the Fourier number. Reprinted from [23], with permission from © The Optical Society.

Based on Equation (5), the reconstructed Raman spectra per cycle at different *Fo*_e_ is shown in Figure 5a. With the increase of *Fo*_e_, the Raman peak is redshifted (softening), the linewidth becomes slightly broader, and the Raman intensity increases significantly. The variations of normalized Raman intensity and Raman shift against *Fo*_e_ are shown in Figure 5b,c. As the temperature increases rapidly at the beginning of laser heating, the normalized Raman intensity and Raman shift decrease quickly correspondingly. With the increase of heating time, these two parameters will reach a constant when the sample reaches steady state. Based on Equation (6), different trial values of thermal diffusivity are used to find the best fitting curve for the experimental data. The thermal diffusivity of the silicon cantilever is determined at 9.17 × 10^−5^ m^2^/s, which is very close to the reference value of 8.66 × 10^−5^ m^2^/s [23]. Similarly, the thermal diffusivity of carbon nanotube fiber is also determined, which is around 1.74 × 10^−5^ m^2^/s [24]. Although this technique has only been used in 1D materials for concept design and testing, it can also be used to characterize the thermal diffusivity of 2D materials, either supported or suspended.

For the TD-Raman technique, neither temperature rise nor laser absorption information are needed. Therefore, it provides higher level physics understanding. However, a technical issue faced in experiment is that, when the heating time is too short, the overall laser on time is very short, and it takes extremely long time to collect the Raman signal. Thus, stage shift or environment noise will increase the uncertainty, which indicates that it is extremely challenging to study very fast thermal transport phenomena. This issue can be resolved using the frequency-resolved Raman (FR-Raman) technique without sacrificing the measurement accuracy.

### 3.2. Frequency-Resolved Raman (FR-Raman)

**Figure 6:**
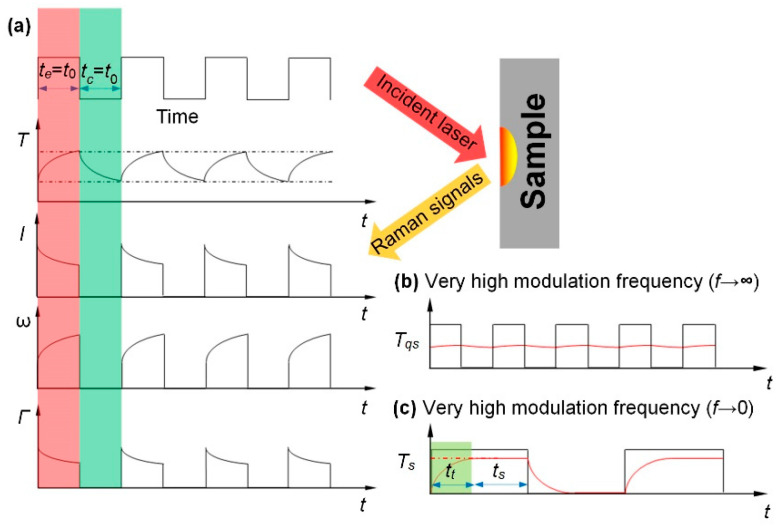
(**a**) Mechanism of frequency-resolved (FR)-Raman. (**b**) Temperature variation at quasi-steady state. (**c**) Temperature variation at very low frequency (close to steady-state). Reprinted from [25], with permission from © The Optical Society.

For the FR-Raman technique, as shown in Figure 6, an amplitude-modulated square-wave laser with different frequencies is used for both sample heating and Raman signals collecting [25]. Figure 6a shows that durations of the laser excitation time and the thermal relaxation time are the same. The temperatures at the beginning and end of the laser excitation time are frequency-dependent. At very high frequencies, shown in Figure 6b, the temperature rise in the laser excitation time and the temperature fall in the thermal relaxation time are almost negligible, that is, the temperature of the sample can be assumed to be constant in the whole process. Furthermore, this state is named as “quasi-steady state”. At very low frequencies, shown in Figure 6c, the laser excitation time is long enough for the temperature rising to a steady-state temperature. Furthermore, the rising period, which was much smaller than the heating period, can be neglected. As a result, the sample temperature can be seen as a constant during the excitation time. Furthermore, this state is termed “steady state”. The temperature rise values at these two states are then taken as ∆*T*_qs_ and ∆*T*_s_, where ∆*T*_qs_ = ∆*T*_s_/2. As the heating effect increases with the decrease of frequency, Raman intensity decreases and Raman peak redshifts. The Gaussian distribution function is used to fit the Raman peaks to obtain precise Raman properties: intensity, Raman shift, and linewidth. The variation of these properties against the modulation frequency can be fitted to determine the thermal diffusivity of a sample. The determined thermal diffusivities of Si based on Raman intensity and Raman shift are 9.57 × 10^−5^ m^2^/s and 11.00 × 10^−5^ m^2^/s, respectively, which agree well with literature value.

**Figure 7:**
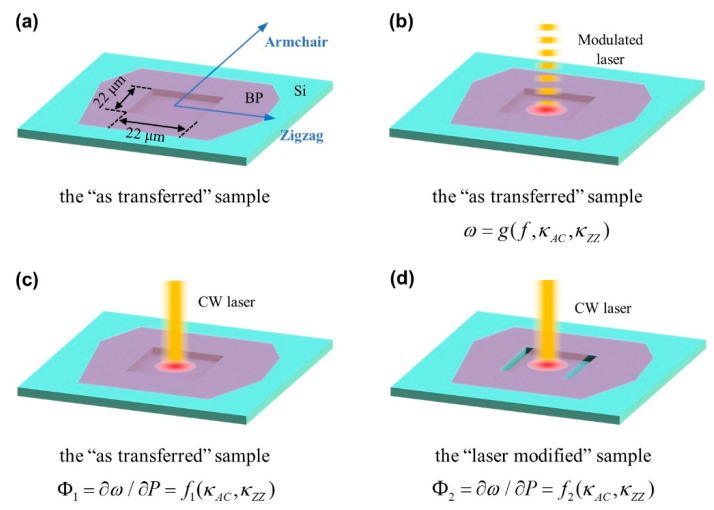
(**a**) Schematic of a suspended black phosphorus (BP) sample. (**b**) Sample irradiated by a modulated laser beam. (**c**) Sample irradiated by a CW laser beam before cutting. (**d**) Sample irradiated by a continuous wave (CW) laser beam after cutting at two parallel boundaries. Reprinted from [26], with the permission of AIP Publishing.

**Figure 8:**
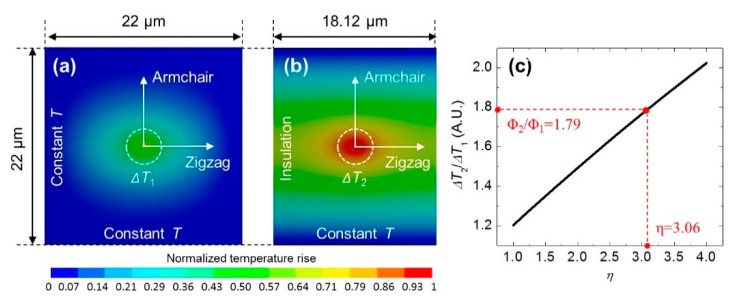
(**a**,**b**) Simulated normalized temperature rise distribution in the 157.6-nm thick sample before cutting and after cutting. (**c**) The theoretical curve of ∆*T*_2_/∆*T*_1_ as a function of *η*. Reprinted from [26], with the permission of AIP Publishing.

The FR-Raman technique not only provides a novel way to probe transient thermal transport with very high temporal resolution, but also can be used to characterize the anisotropic thermal conductivities of materials without the need of optical absorption and temperature coefficient [26]. The example given here is for the measurement of black phosphorus (BP). As shown in Figure 7a, the armchair and zigzag directions of the suspended BP are aligned along an edge of a square dent. Figure 7b shows that the sample is irradiated by a modulated laser beam and the corresponding Raman spectra are collected. Then, as shown in Figure 7c,d, Raman spectra of the sample before and after cutting are collected upon continuous wave (CW) laser irradiation with different laser powers. The power differential of the Raman shift Φ_1_ and Φ_2_ are determined. For these two parameters, Φ_1_ depends on the armchair thermal conductivity *κ*_AC_ and the zigzag thermal conductivity *κ*_ZZ_, while Φ_2_ mainly depends on *κ*_AC_. These two values are linearly related to the average temperature rise in the heating region ∆*T*_1_ and ∆*T*_2_, which are obtained from the ANSYS simulation results shown in Figure 8a,b. Afterwards, as shown in Figure 8c, *η* (*κ*_ZZ_/*κ*_AC_) can be determined by interpolating Φ_2_/Φ_1_ to the simulated curve of ∆_2_/∆*T*_1_ and *η*.

**Figure 9:**
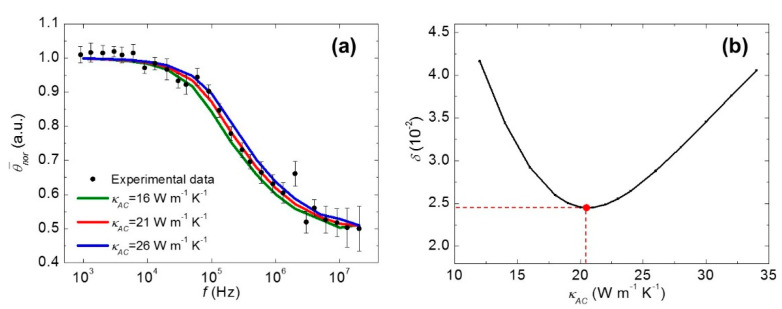
(**a**) The various theoretical fitting curves of experimental data. (**b**) The standard deviation *δ* of experimental data to theoretical curves as a function of *κ*_AC_. Reprinted from [26], with the permission of AIP Publishing.

The determination of *κ*_AC_ is realized by comparing the experimental normalized average temperature rise $\Delta {\bar{T}}_{nor\_e}$ with its simulated counterpart $\Delta {\bar{T}}_{nor\_s}$. In the simulation, *κ*_AC_ is adjusted to reach a minimum standard deviation between $\Delta {\bar{T}}_{nor\_e}$ and $\Delta {\bar{T}}_{nor\_s}$. Furthermore, $\Delta {\bar{T}}_{nor\_e}$ is calculated from *ω*(*f*) using the equation below:

(7) $$\Delta {\bar{T}}_{nor\_e}=1+C\cdot \left[\omega (f)-\omega_{s}\right]/\Delta \omega ,$$

where ∆*ω* and *ω_s_* are obtained from steady state, *C* is a correction coefficient. Theoretically, $\Delta {\bar{T}}_{nor\_e}$ decreases from 1 (steady state) to 0.5 (quasi-steady state), and the experimental data are shown in Figure 9a. ANSYS is also used to simulate the thermal response to determine $\Delta {\bar{T}}_{nor\_e}$, which is equal to:

(8) $$\Delta {\bar{T}}_{nor\_s}=\frac{\int_{0}^{1/2f}\sum_{n=0}^{\infty }{\left(-1\right)}^{n}\cdot \Delta T_{s}(t_{P}+n/(2f))dt}{(1/2f)\cdot \Delta {\bar{T}}_{\infty }},$$

where $\Delta T_{s}(t)$ represents the temperature rise as a function of time, $\Delta {\bar{T}}_{\infty }$ is the average temperature rise in the steady state. Since *η* is already known, $\Delta {\bar{T}}_{nor\_s}$ is a function of *f* and *κ*_AC_. Thus, the variation of $\Delta {\bar{T}}_{nor\_s}$ against *f* is only determined by *κ*_AC_. Figure 9a shows three fitting curves by adjusting the *κ*_AC_ values. Based on the curve between *κ*_AC_ and standard deviation *δ*, shown in Figure 9b, the best fitting value is obtained.

### 3.3. Frequency-Domain Energy Transport State-Resolved Raman (FET-Raman)

**Figure 10:**
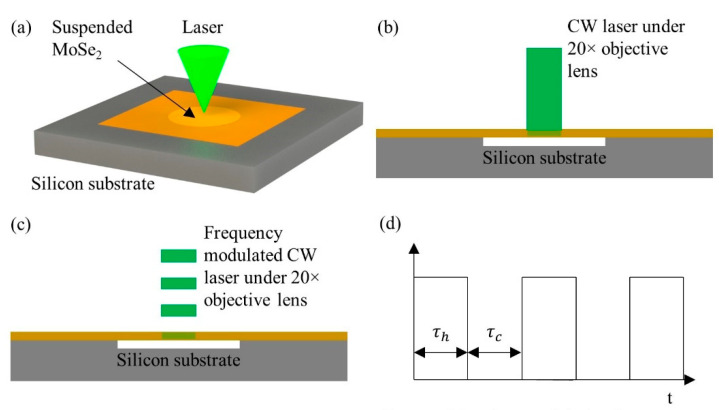
(**a**) Schematic of suspended MoSe_2_ nanosheet. (**b**) Suspended MoSe_2_ sample under CW laser and (**c**) under frequency-modulated CW laser heating and Raman excitation. (**d**) Square wave used to modulate the CW laser. Reprinted from [27], with permission from Elsevier, 2019.

For FR-Raman, the data fitting is for the Raman shift against the modulation frequency, and it takes quite tremendous measurements. An alternative, named FET-Raman, is to fix the frequency, but vary the laser power and study the Raman shift change against laser power [27,28]. Here, we take the work on MoSe_2_ to introduce this technique. The physical principle of this technique is shown in Figure 10. During each heating period, since the thermal diffusion length of MoSe_2_ in the cross-plane direction is much longer than the sample thickness, the thermal transport in the cross-plane direction can be neglected. The first energy transport state is the steady state heating constructed by a CW laser, shown in Figure 10b. By using different laser powers (*P*), a parameter named Raman shift power coefficient (RSC) is obtained: $\psi_{\mathrm{CW}}=\partial \omega /\partial P=\alpha \cdot (\partial \omega /\partial T)\cdot f_{1}(\kappa )$, where *α* is laser absorption coefficient, ∂ω/∂T is Raman shift temperature coefficient, and κ is in-plane thermal conductivity of MoSe_2_. The second energy transport state is a transient state heating constructed by a square wave modulated CW laser, shown in Figure 10c,d. After a sufficient number of heating cycles, the sample temperature will vary periodically with time. As illustrated in Section 3.2, the energy transport state changes from quasi-steady state to steady state with the decrease of frequency. Therefore, an appropriate frequency should be selected to construct a transient state with good sensitivity. Based on the curves shown in Figure 9a, this frequency should be around the middle of quasi-steady state to steady state range. Then, a similar RSC value is also obtained: $\psi_{\mathrm{FR}}=\partial \omega /\partial P=\alpha \cdot (\partial \omega /\partial T)\cdot f_{2}(\kappa ,\rho c_{p})$, where $\rho c_{p}$ is volumetric heat capacity of the sample. 

**Figure 11:**
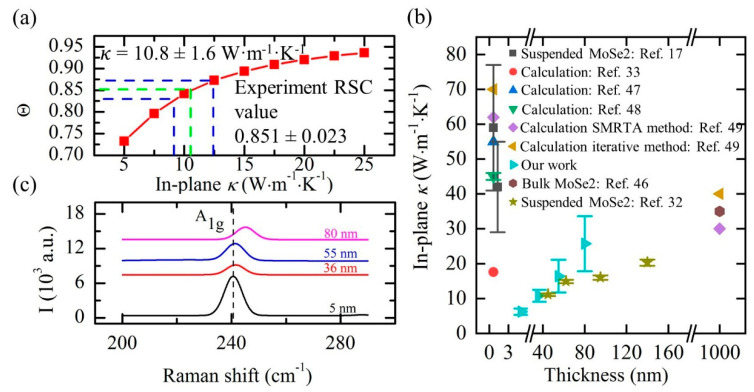
(**a**) 3D numerical simulation of a 36 nm-thick MoSe_2_ sample. (**b**) Comparison of in-plane *κ* values of MoSe_2_ nanosheets from different studies. (**c**) The blue shift of Raman peak with the increase of sample thickness. Reprinted from [27], with permission from Elsevier, 2019.

As the thermal diffusion lengths in the two states are different, a dimensionless normalized RSC $\Theta =\psi_{\mathrm{FR}}/\psi_{\mathrm{CW}}=f_{3}(\kappa ,\rho c_{p})$ is used to completely eliminate the effects of *α* and ∂ω/∂T. In the experiment, very low laser powers are employed to ensure a moderate temperature rise of the sample. This ensures the effect of volumetric heat capacity change with temperature can also be ignored. That is, the normalized RSC is only related to the in-plane thermal conductivity of MoSe_2_. A three-dimensional (3D) numerical modeling is then conducted to characterize the temperature profile under these two states. A theoretical relation between the ratio of temperature rises in the two states and in-plane thermal conductivity is obtained. The in-pane thermal conductivity of the sample can be determined by interpolating the experimental data into the curve. As shown in Figure 11a, κ of a 36 nm-thick MoSe_2_ is determined as 10.8 ± 1.6 W·m^−1^·K^−1^. Figure 11b shows the κ values of MoSe_2_ with different thickness from different studies, and the feasibility of FET-Raman technique firmly verified. Figure 11c shows the blue shift of Raman spectra with the increased thickness, which means the interlayer Van der Waals force in MoSe_2_ is increasing. Additionally, combining with the TET technique, the FET-Raman can also be used to characterize the anisotropic thermal conductivity of carbon fibers [28].

## 4. Energy Transport State-Resolved Raman

Similar to the modulation of laser in TD-Raman technique, the CW laser used in FET-Raman technique is also modulated with a square wave. In addition to the modulation in time domain, this energy transport design can also be extended to spatial domain to control the energy transport states. Yuan et al. reports a novel technique for non-contact simultaneous determination of interface thermal resistance (*R*) and hot carrier diffusion coefficient (*D*) of MoS_2_ nanosheets on c-Si by varying the laser heating area [29]. In this work, the constructed two energy transport states are in spatial domain. To be fully free from the large errors of laser absorption evaluation and temperature coefficient calibration, a further developed technique named energy transport state-resolved Raman (ET-Raman) is developed to determine *R* and *D* [30,31]. In this technique, three distinct energy transport states in both spatial and time domains are constructed to probe materials’ thermal response. Furthermore, a five-state ET-Raman technique is proposed to measure *κ* of MoS_2_, and the effects of *R* and *D* are taken into consideration, and all these properties are determined simultaneously [32].

**Figure 12:**
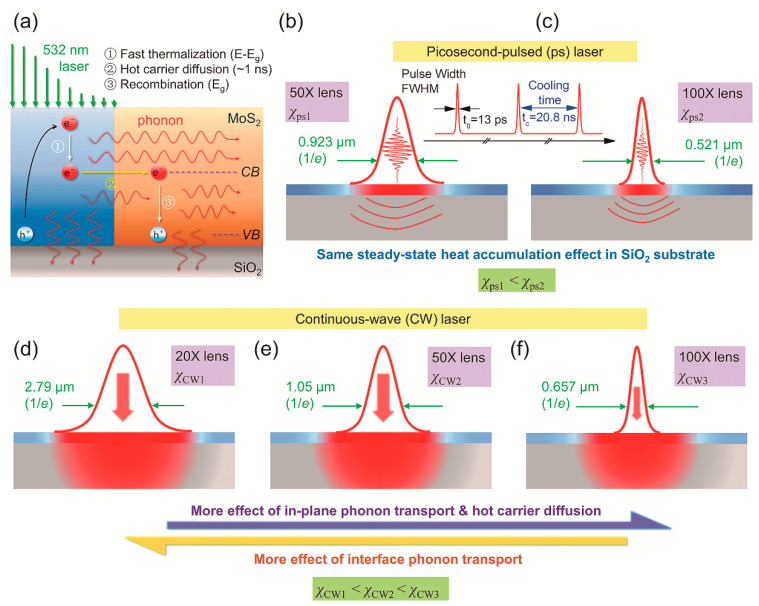
The mechanism of five-state energy transport state-resolved Raman (ET-Raman) technique. (**a**) Physical processes happening in MoS_2_ upon laser irradiating. (**b**,**c**) Two transient states in picosecond laser heating under 50× and 100× objective lenses. (**d**–**f**) Three steady states under a CW laser with 20×, 50×, and 100× objective lenses. Reproduced from [32], with permission of the PCCP Owner Societies.

Figure 12a shows the physical principles of this technique. A laser with 532 nm wavelength is used to irradiate the sample for both laser heating and Raman probing. As the excitation energy is higher than the band gap of MoS_2_, three physical processes take place. First, hot carriers are generated, and then diffuse in space before the electron-hole recombination. Subsequently, phonons, which receive energy from the hot carriers or electron-hole recombination, transports the energy by heat conduction. This process mainly depends on *κ* of the sample. The third process, which is determined by *R*, is the heat conduction from MoS_2_ to the substrate. As shown in Figure 12d–f, combined with three different objective lenses (20×, 50×, and 100×), a CW laser is used to construct three steady states. Similar to FET-Raman, three RSCs ($\chi_{{\mathrm{CW}}_{1}}$, $\chi_{{\mathrm{CW}}_{2}}$, $\chi_{{\mathrm{CW}}_{3}}$) can be obtained, and we have $\chi_{{\mathrm{CW}}_{3}}>\chi_{{\mathrm{CW}}_{2}}>\chi_{{\mathrm{CW}}_{1}}$. With the decrease of laser spot size, *D* and κ play a much more important role in determining the temperature of the sample. Based on this, the effects of *R*, κ, and *D* can be differentiated.

In this technique, two transient states are designed by using a picosecond-pulsed laser with two objective lenses (50× and 100×), shown in Figure 12b,c, to rule out the large errors introduced by laser absorption evaluation and temperature coefficient calibration. Similarly, two RSCs under the two lenses are obtained as $\chi_{{\mathrm{ps}}_{1}}$ and $\chi_{{\mathrm{ps}}_{2}}$, respectively. Furthermore, the heat accumulation effect can be ruled out by taking the difference of these two RSCs as $\chi_{{\mathrm{ps}}_{2}}-\chi_{{\mathrm{ps}}_{1}}$. Based on the five measured RSCs, three dimensionless normalized RSCs $\Theta_{1}=\chi_{{\mathrm{CW}}_{1}}/\left(\chi_{{\mathrm{ps}}_{2}}-\chi_{{\mathrm{ps}}_{1}}\right)$, $\Theta_{2}=\chi_{{\mathrm{CW}}_{2}}/\left(\chi_{{\mathrm{ps}}_{2}}-\chi_{{\mathrm{ps}}_{1}}\right)$, and $\Theta_{3}=\chi_{{\mathrm{CW}}_{3}}/\left(\chi_{{\mathrm{ps}}_{2}}-\chi_{{\mathrm{ps}}_{1}}\right)$ are obtained. Furthermore, all these coefficients, which are related to the temperature rise of the sample, are functions of $\rho c_{p}$, *R*, *D*, and κ.

**Figure 13:**
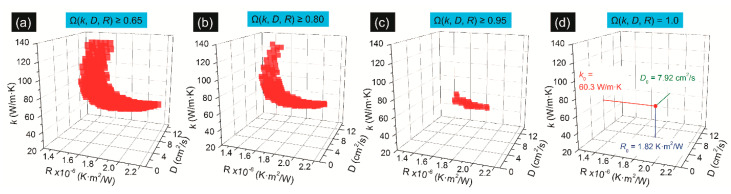
The normalized probability distribution function Ω(κ,D,R) with the probability of 0.65 in (**a**), 0.80 in (**b**), 0.95 in (**c**), and 1.0 in (**d**). Reproduced from [32], with permission of the PCCP Owner Societies.

A 3D numerical modeling is employed to calculate the temperature rise to determine *R*, *D*, and κ simultaneously. To normalize the (*κ*, *D*, *R*) space data, a normalized probability distribution function $\Omega_{i}=\exp\left[-{\left(\Theta_{i}-\Theta_{\exp\_i}\right)}^{2}/\left(2\sigma_{i}^{2}\right)\right]$ (*i* = 1, 2, and 3) is employed. Furthermore, $\Theta_{i}$ and $\Theta_{\exp\_i}$ are normalized RSCs from 3D modeling and experiment, respectively. $\sigma_{i}$ is the experimental uncertainty. Then, (*κ*, *D*, *R*) of the sample can be determined when a composite probability distribution function $\Omega \left(\kappa ,D,R\right)=\Omega_{1}\cdot \Omega_{2}\cdot \Omega_{3}$ is equal to 1. Figure 13 shows the determination of the three parameters for 2.4 nm-thick MoS_2_. With the increase of probability level from 0.65 to 1.0, the (*κ*, *D*, *R*) space range is decreased to have only one point in the space that could give Ω(κ,D,R)=1. Based on this, there three parameters can be obtained as $\kappa_{0}=60.3\;W\cdot m^{-1}\cdot K^{-1}$, $D_{0}=7.92{\;\mathrm{cm}}^{2}\cdot s^{-1}$, and $R_{0}=1.82\times 10^{-6}\;K\cdot m^{2}\cdot K^{-1}$, respectively.

In addition to supported 2D materials, the ET-Raman technique can also be used for suspended 2D materials. However, a strong heat accumulation will happen in suspended samples because of the very short pulse interval for the picosecond laser, a nanosecond laser is used instead [33,34]. Wang et al. used one CW laser and one nanosecond laser with the same wavelength to construct the steady state heating and transient state heating [33]. The in-plane thermal conductivities of suspended MoS_2_ and MoSe_2_ with different thickness are measured. However, the hot carrier effect is not considered in this work. Zobeiri et al. developed a three-state nanosecond ET-Raman technique to measure *κ* and *D* of nm-thick suspended WS_2_ films [34].

**Figure 14:**
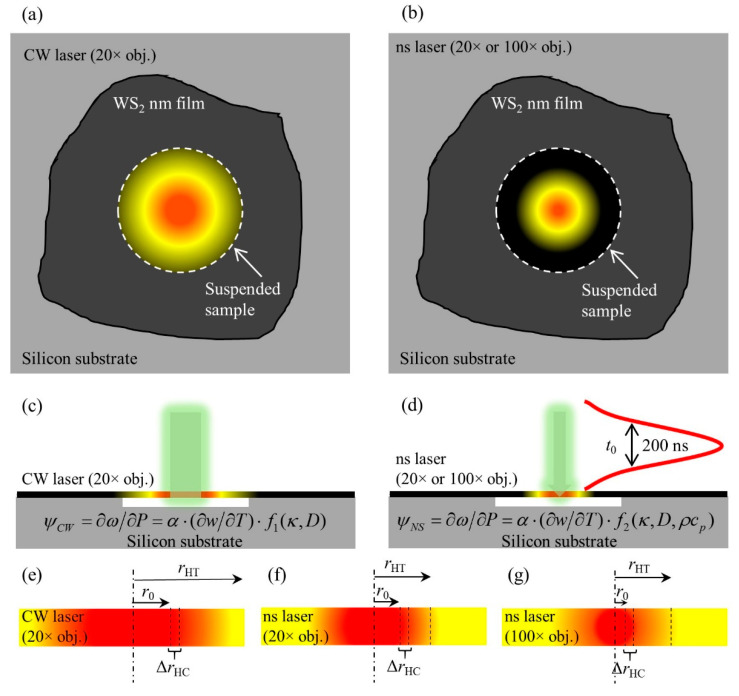
(**a**,**b**) Heat conduction of suspended WS_2_ nanosheets irradiated by a CW and a nanosecond laser. (**c**,**d**) Steady state and transient state constructed using the two lasers. (**e**–**g**) Heat diffusion length, laser spot radius, and hot carrier diffusion length under the three states. Reprinted from [34], with permission from Elsevier, 2019.

In the three-state nanosecond ET-Raman technique, the three heat transport states are constructed with two lasers and two objective lenses. As shown in Figure 14a, a CW laser with a 20× objective lens is used to construct the steady state. Figure 14b shows that two transient states are constructed using a nanosecond pulsed laser and two different objective lenses (20× and 100×). Since the thickness is very thin, the temperature distribution in the thickness direction is assumed to be uniform. Similarly, the three RSCs under the three states are obtained as *ψ_CW_*, *ψ_ns_*_20_, and *ψ_ns_*_100_. As shown in Figure 14c, *ψ_CW_* is a function of *α*, *κ*, *D*, and Raman temperature coefficient (∂ω/∂T). Meanwhile, both *ψ_ns_*_20_ and *ψ_ns_*_100_ are a function of *α*, *κ*, *D*, *ρc_p_*, and ∂ω/∂T, shown in Figure 14d. Furthermore, the effects of *κ* and *D* can be distinguished by using the two objective lenses to vary the local heating size. Considering the moderate temperature rise in the experiment, *ρc_p_* can be taken as a constant.

**Figure 15:**
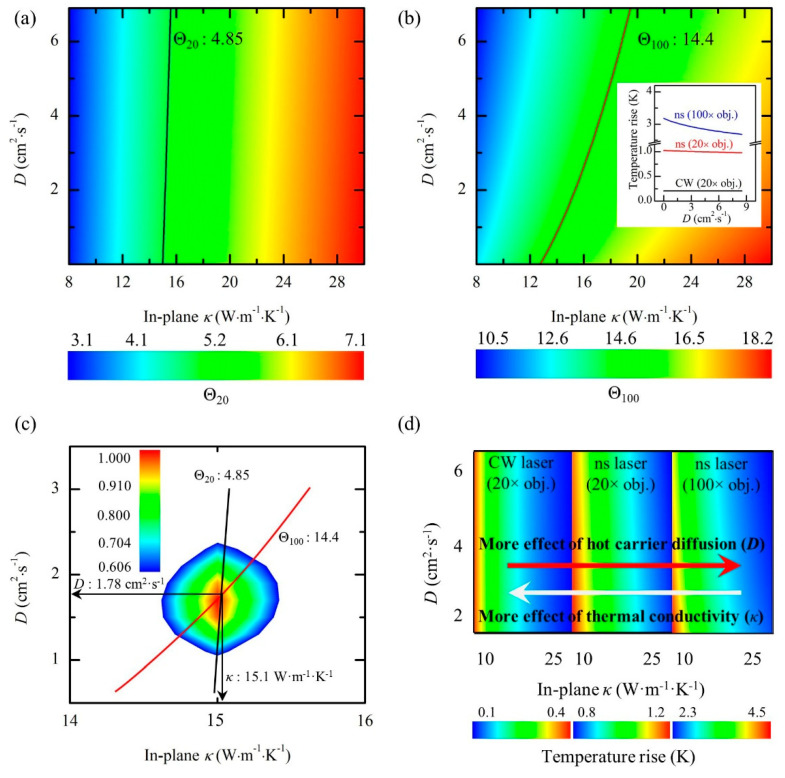
(**a**,**b**) Θ for different *κ* and *D* values obtained from simulation and experiments of the 13 nm-thick sample under (**a**) 20× and (**b**) 100× objective lenses. (**c**) Measured *κ* and *D*. (**d**) The sensitivity variation of temperature rise to *κ* and *D* under different energy transport states. Reprinted from [34], with permission from Elsevier, 2019.

Based on the three RSCs, two normalized RSCs are defined as $\Theta_{20}=\psi_{ns20}/\psi_{CW}$ and $\Theta_{100}=\psi_{ns100}/\psi_{CW}$. Then, the effects of *α* and ∂ω/∂T are ruled out. Figure 14e–g shows the heat and hot carrier diffusion lengths in the in-plane direction of suspended sample. Under steady state, the heat can transfer to the boundaries of the sample. While under the two transient states, the thermal transport is nearly confined in the laser spot area. That is, the effect of *κ* on thermal transport is more significant under steady state. As shown in Figure 15d, the effect of *κ* becomes less significant with the decrease of local heating size, while the effect of *D* becomes more prominent with the decrease of local heating size. The temperature rise under the three states are simulated to obtain the theoretical Θ values under different *κ* and *D* trial values, shown in Figure 15a,b. The solid lines indicate that several (*κ*, *D*) combinations can match the experimental values. As shown in Figure 15c, by using the two solid lines to locate the cross-point, *κ* and *D* values are determined as 15.1 W·m^−1^·K^−1+^ and 1.78 cm^2^·s^−1^, respectively.

In summary, different energy transport states are constructed in both time and space domains to characterize the thermal properties of supported or suspended samples. Either a picosecond or a nanosecond laser is used to realize the differential in time domain. Similarly, the TDTR technique measures thermal properties by heating the surface of the sample with a train of laser pulses and detecting the resulting temperature variation through the reflectivity of the surface with a time-delayed laser. This technique is able to detect temperature evolution at micrometer-scale and picosecond-scale resolutions, which indicates that it can be used to explore non-equilibrium thermal phenomena [14]. The ET-Raman technique in fact measures a material’s thermal response within a pulse in an integral way. It gives an average temperature within a very short time domain (ns or ps), and provides a completely new way to characterize nanoscale energy transport.

## 5. Probing of Conjugated Hot Carrier Transport

**Figure 16:**
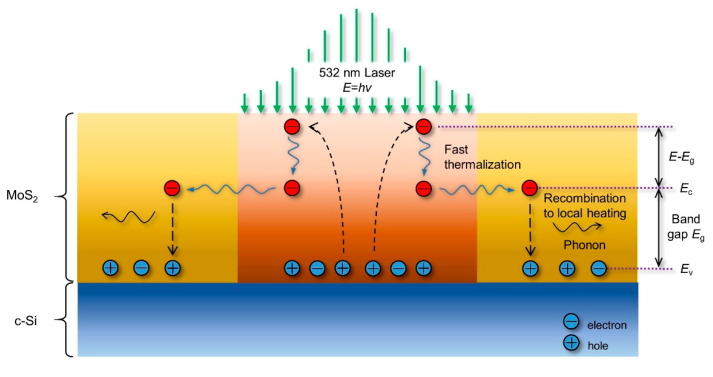
The physical process of hot carrier diffusion. Reproduced from [29], with permission from The Royal Society of Chemistry.

In most of the work on Raman study of energy transport in 2D materials, hot carrier diffusion is not considered, although this effect could be critically important, especially for tightly focused laser spot (<0.5 µm diameter). Figure 16 shows the physics of hot carrier diffusion. The sample is irradiated by a laser, the energy of which is higher than the bandgap of MoS_2_. Thus, electrons are excited to the conduction band while leaving holes in the valence band. Then a fast thermalization process (about 10^−12^ s) happens, and hot carriers dissipate part of the energy to other electrons and lattice. This process is neglected due to the very short time. The second process is hot carrier diffusion, in which the remaining photon energy carried by electrons is diffused out of the laser spot area before recombining with holes. As this process is typically in nanoseconds, it should be taken into consideration. Afterwards, electrons and holes recombine because of Coulomb attraction, the energy is released by exciting phonons at the same time. The phonons then dissipate the energy with the sample and through layers down to the substrate.

For steady state, the generation and diffusion of heat and hot carriers are governed by two partial differential equations. The first one is the carrier diffusion equation to determine the hot carrier concentration ΔN(r,t) (cm^−3^):

(9) $$D\nabla^{2}\Delta N-\frac{\Delta N}{\tau }+\frac{\partial n_{0}}{\partial T_{\mathrm{CW}}}\frac{\Delta T_{\mathrm{CW}}}{\tau }+\Phi \alpha =0,$$

where *D* (cm^2^·s^−1^) is carrier diffusion coefficient, *τ* (s) is electron-hole recombination time of the sample, Φ (photons per cm^3^ per s) is incident photon flux of the laser source, and *n*_0_ (cm^−3^) is the equilibrium free-carrier density at temperature *T*. The second equation is the thermal diffusion equation which involves the free carrier density:

(10) $$\kappa \nabla^{2}\Delta T_{\mathrm{CW}}+(h\nu -E_{g})\Phi \alpha +\frac{E_{g}\Delta N}{\tau }=0,$$

where ΔT(r,t) (K) and *E_g_* (eV) are temperature rise and bandgap energy of the sample, and *hν* is photon energy of the laser source. Due to the hot carrier diffusion effect, the real heating area will be larger than the laser irradiating area, and is highly related to the hot carrier diffusion length ($L_{D}=\sqrt{\tau D}$). As a result, when the laser spot size is large enough, the hot carrier diffusion will have negligible effect on the heating area.

For transient state, Yuan et al. used a picosecond laser to characterize the thermal transport for supported samples [32]. The laser pulse (13 ps) is so short that the heat conduction becomes very weak. Then, five transport states in both time and space domains are constructed. *κ* and *R* values are determined by taking *D* into consideration. Zobeiri et al. used a nanosecond laser to study the thermal transport for suspended samples [34]. Three transport states in both time and space domains are constructed. *κ* of the suspended sample is determined by taking *D* into consideration. As shown in Figure 15a, due to the large laser spot size under 20× objective lens, the effect of *D* on *κ* of the sample is very tiny. While under 100× objective lens, shown in Figure 15b, due to the relatively small laser spot size, the effect of *D* cannot be neglected.

## 6. Probing of Thermal Nonequilibrium among Phonon Branches

The physical process happening inside different Raman-based methods consists of energy transfer among photons, electrons, and phonons. For phonons, three optical branches, which are the longitudinal optical (LO), transverse optical (TO), and flexural optical (ZO) branches, are included. Similarly, there are also three acoustic branches (LA, TA, and ZA). Furthermore, the temperatures of these branches are at nonequilibrium under laser excitation. ZA phonons are the main heat carriers in the heat conduction process, while optical phonons are the ones probed by Raman spectroscopy. Thus, neglect of nonequilibrium between ZA phonons and optical phonons can induce significant underestimation of thermal conductivity. Wang et al. designed and employed a nanosecond ET-Raman technique to explore the temperature nonequilibrium among different phonon branches [35].

**Figure 17:**
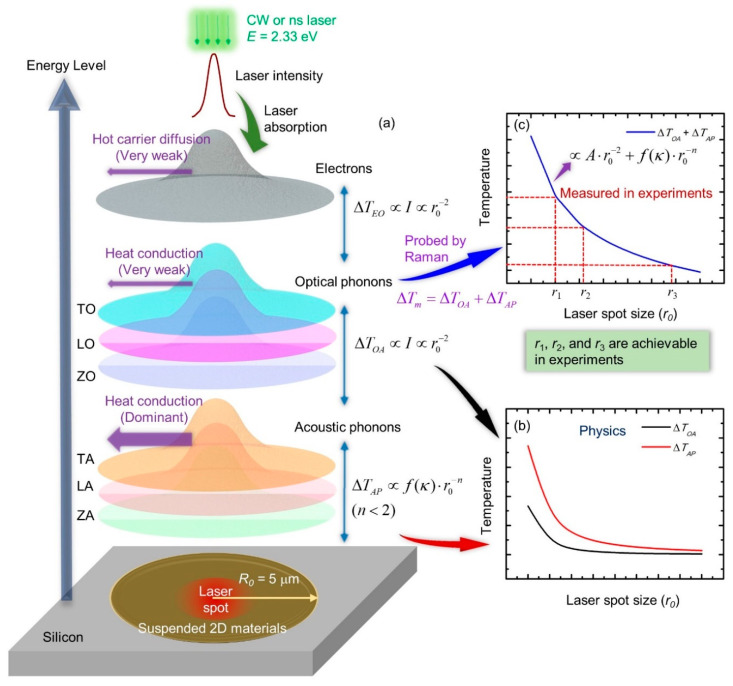
(**a**) The energy transfer process among different energy carriers in 2D materials under laser irradiation. (**b**) The temperature difference between optical phonons and acoustic phonons against laser spot size. (**c**) The determination of thermal conductivity and energy coupling coefficient between optical phonons and acoustic phonons. Figure reproduced from [35], with permission from John Wiley and Sons.

Figure 17a shows the energy transfer process among different energy carriers. Optical phonons (OP) receive energy from hot carriers, and will have a prominent temperature rise. Then, OP will transfer majority of the energy to acoustic phonons (AP) through energy coupling. For the temperature difference between OP and AP, we have $\Delta T_{\mathrm{OA}}\propto I\propto r_{0}^{-2}$, where *I* and *r*_0_ are the laser energy and radius of laser spot. Furthermore, the temperature rise of AP ($\Delta T_{\mathrm{AP}}$) is related to both *r*_0_ and *κ*, we have $\Delta T_{\mathrm{AP}}\propto f(\kappa )\cdot r_{0}^{-n}$ with *n* < 2. As shown in Figure 17b, with the increase of laser spot size, $\Delta T_{\mathrm{OA}}$ decreases to zero faster than $\Delta T_{\mathrm{AP}}$, which indicates that the effect of energy coupling between OP and AP is negligible under very large laser spot. In Raman-based techniques, as shown in Figure 17c the temperature rise of OP, which can be expressed as $\Delta T_{m}=\Delta T_{\mathrm{OA}}+\Delta T_{\mathrm{AP}}\propto Ar_{0}^{-2}+f(\kappa )\cdot r_{0}^{-n}$, is probed under different laser spot size. Afterwards, the percentages of $\Delta T_{\mathrm{OA}}$ and $\Delta T_{\mathrm{AP}}$ in $\Delta T_{m}$ are determined. 

**Figure 18:**
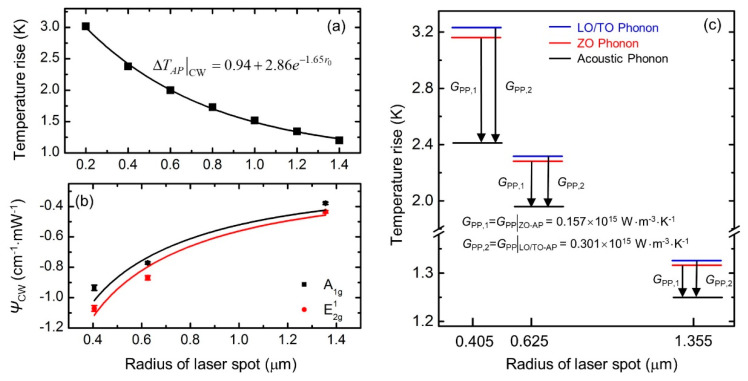
Determination of coupling factor of MoS_2_ under steady state. (**a**) Temperature rise of acoustic phonons obtained from simulation. (**b**) Experimental values and fitting curves of *ψ*_CW_ against laser spot size. (**c**) Distinct temperatures of different phonon branches. Figure reproduced from [35], with permission from John Wiley and Sons.

In nanosecond ET-Raman experiments, the measured *ψ* values are linearly related to Raman intensity weighted temperature rise of the sample. The Raman intensity weighted temperature rise measured under steady state can be written as:

(11) $$\Delta {{\bar{T}}_{m}|}_{\mathrm{CW}}=\Delta {{\bar{T}}_{\mathrm{AP}}|}_{\mathrm{CW}}+\frac{1}{3}\cdot \frac{I_{0}}{\tau_{L}}\cdot \frac{\delta }{{G_{\mathrm{pp}}|}_{\mathrm{CW}}},$$

where ${G_{\mathrm{pp}}|}_{\mathrm{CW}}$ is the energy coupling factor between OP and AP, *I*_0_ is the absorbed laser power per unit area at the center of laser spot, *τ*_L_ is the laser absorption depth, and *δ* (0 < *δ* < 1) is portion of laser energy transferred from the measured Raman mode optical phonons to acoustic phonons. Figure 18a shows the variation of ${\Delta {\bar{T}}_{\mathrm{AP}}|}_{\mathrm{CW}}$ against laser spot size using a 3D numerical modeling for the 55 nm thick MoS_2_. Based on this, the relation between *ψ*_CW_ and ${\Delta {\bar{T}}_{m}|}_{\mathrm{CW}}$ can be expressed as:

(12) $$\psi_{\mathrm{CW}}=A\cdot \left[(0.94+2.86e^{-1.65r_{0}})+\frac{1}{3}\cdot \frac{P}{\pi r_{0}^{2}\tau_{L}}\cdot \frac{\delta }{{G_{\mathrm{pp}}|}_{\mathrm{CW}}}\right]/P,$$

where *A* is determined by Raman shift temperature coefficient and laser absorption, *P* is the laser power, *r*_0_ is the radius of laser spot. Figure 18b shows the *ψ*_CW_ values under three objective lenses, and Equation (12) is used to obtain *ψ*_CW_~*r*_0_ fitting curve. Then, the energy coupling factors between OP and AP for the two Raman modes under steady state are determined as 0.301 × 10^15^ W·m^−3^·K^−1^ for $E_{2g}^{1}$ mode and 0.157 × 10^15^ W·m^−3^·K^−1^ for A_1g_ mode. Afterwards, the percentages of $\Delta T_{\mathrm{OA}}$ and $\Delta T_{\mathrm{AP}}$ can be distinguished, and the temperatures of LO/TO phonon, ZO phonon, and AP are obtained, shown in Figure 18c. Specifically, the temperature difference between OP and AP takes more than 25% of the measured temperature rise under a small laser spot size. Thus, $\Delta T_{\mathrm{OA}}$ cannot be neglected when a small laser spot is used.

However, for the FR-Raman and TD-Raman, this effect is ruled out since they only use the Raman shift change versus modulation frequency. The phonon branch temperature difference is a constant and has no effect in the physical data processing. Furthermore, in other techniques, like the TET technique, the phonon branch temperature difference is negligible. In TET, we fit the trend of the temperature change against time to determine the thermal diffusivity, then determine the thermal conductivity. The electrons-OP and OP-AP temperature difference will only add a constant value on the AP temperature, and does not affect the fitting results. Note in the TET technique, since the heating is over the whole sample and the inter-phonon branch heat current is significantly lower than the laser intensity in this work, the electron temperature is very close to that of the AP and their temperature difference is negligible compared with the measured temperature rise. The temperature difference between electron and AP can be calculated by $T_{e}-T_{AP}=I^{2}RV^{-1}\left[{\left(\sum G_{\mathrm{ep}}\right)}^{-1}+{\left(\sum G_{\mathrm{pp}}\right)}^{-1}\right]$, where *I* is the current flowing through the sample, *R* is its resistance at steady state, *V* is the volume of the sample, *G*_ep_ is the coupling factor between electrons and OP. As there are three phonon branches for both OP and AP, here the sums of all the corresponding coupling factors are used in the calculation. For instance, the in-plane thermal conductivity of graphene paper is obtained as 634 W·m^−1^·K^−1^ using the TET technique. The length, width, and thickness of the sample are around 17 mm, 0.28 mm, and 28.6 μm, respectively [36]. Then, based on the coupling factors, the temperature difference between electrons and AP is calculated to be around 2.8 × 10^−8^ K, which is negligible compared with a measured temperature rise of 2 K [35,37].

## 7. Concluding Remarks and Outlooks

As Raman spectroscopy can be used to characterize the energy and charge transport in 2D materials, many different Raman-based techniques have been developed over the last decade. Steady state Raman can be used to measure the thermal conductivity and interface thermal resistance. However, both temperature calibration and laser absorption measurement, which induce large errors, are needed. To overcome this drawback, techniques involving time resolving, which include TD-Raman and FR-Raman, are proposed. For TD-Raman, it is not appropriate for studying very fast thermal transport phenomena. Though FR-Raman can be used for fast thermal transport, a large number of measurements under different frequencies are required for the data fitting process. Then, FET-Raman technique, with a fixed frequency, was developed to characterize the thermal properties by studying the Raman shift change against laser power.

For all the Raman-based techniques mentioned above, the hot carrier diffusion effect is not considered. By constructing different energy transport states in both time and space domains, ET-Raman techniques are proposed. For supported samples, a picosecond laser and a CW laser are combined to realize the simultaneous measurement of *κ*, *D*, and *R*. To reduce the heat accumulation effects in suspended samples, a nanosecond laser and a CW laser are used together to measure *κ* and *D*. As all Raman-based techniques share the similar energy transport process, the neglect of temperature nonequilibrium among different energy carriers can also introduce errors in the thermal property characterization. The nanosecond ET-Raman technique is further developed to study the energy coupling between OP and AP. The corresponding coupling factors are determined, and a much more accurate thermal conductivity is also obtained. This breakthrough is expected to move the Raman-based energy transport probing to an unprecedented level.

In summary, Raman-based techniques show excellent suitability and performance in characterizing the energy and charge transport of 2D materials. Additionally, since 2D materials are extremely thin, the beam scattering techniques (e.g., XRD) cannot obtain sound diffraction signal and determine the in-plane lattice size. On the other hand, using thermal diffusivity (*α*) measured by Raman spectroscopy, we can measure the thermal diffusivity at different temperatures. Then by using the thermal reffusivity theory, we can extend to obtain the thermal reffusivity at the 0 K limit, and obtain the structure domain size. The thermal reffusivity model of phonons is expressed as:

(13) $$\Theta =\frac{1}{\alpha }=\frac{3}{\nu^{2}}\left(\frac{1}{\tau_{\mathrm{phonon}}}+\frac{1}{\tau_{\mathrm{defect}}}\right)=\Theta_{0}+C\cdot e^{-B/T},$$

where *ν* is the average group velocity, *τ*_phonon_ and *τ*_defect_ are the electron-phonon scattering time and defect scattering time, respectively. $\Theta_{0}$ is the thermal reffusivity at the 0 K limit, and is entitled as residual thermal reffusivity, *B* is a constant proportional to the material’s Debye temperature. Based on Equation (13), Θ decreases with the decrease of temperature and reaches $\Theta_{0}$ at the 0 K limit. Furthermore, the defect scattering intensity from grain boundary, lattice imperfections, chemical impurities, rough edges, and amorphous structures, etc. can be reflected by $\Theta_{0}$. In addition, the lattice vibration also weakens and the phonon population decreases as temperature goes down. From Equation (13), $\Theta_{0}$ can be written as $\Theta_{0}=3/(\nu l_{0})$, where *l*_0_ is the mean free path limited by defect scattering. *l*_0_ is called structure thermal domain size, which is actually an effective domain size combining the effect from three-dimensional crystallite [38,39,40].

During Raman scattering of 2D materials, the intensity in fact reflects some critical properties of the 2D materials, like electron excitation energy and interface spacing [22]. In TMDs, based on the light scattering theory and time-dependent perturbation theory, the Raman intensity can be written as:

(14) $$I\propto {\left|\frac{1}{\left[E(T)-E_{i}-i\Gamma (T)\right]\left[E(T)-E_{s}-i\Gamma (T)\right]}\right|}^{2},$$

where *E*(*T*) and *Γ*(*T*) are exciton’s temperature dependent transition energies and damping constants, respectively. *E*_i_ and *E*_s_ are the energy of incident and scattered lights. Based on this equation, the sample temperature will affect electronic band structure, and the corresponding Raman intensity will then be influenced. In addition, the optical properties of the sample and their variation with temperature also affect the Raman intensity. For supported samples, there is an interface spacing between the sample and the substrate. The Raman intensity is altered due to the multi-reflections in this spacing air gap layer. Thus, the interpretation of temperature dependent Raman intensity should take all the factors above into consideration, and it is a critical direction that needs to be explored.

Raman-based techniques are also widely used for exploring the thermal properties of monolayer 2D materials. Guo et al. measured the thermal conductivity of strained monolayer graphene by using optothermal Raman method [41]. Cai et al. measured the thermal conductivity and thermal expansion coefficient of suspended monolayer boron nitride by using optothermal Raman method [42]. Yalon et al. measured the temperature-dependent thermal boundary conductance of monolayer MoS_2_ with AlN and SiO_2_ using Raman thermometry technique [43]. However, the radiative electron-hole recombination effect, which significantly affects the measurement accuracy, is not considered in current Raman-based techniques. Further work should consider this effect and significantly advance the understanding.
